# PTSD: from neurobiology to pharmacological treatments

**DOI:** 10.3402/ejpt.v7.31858

**Published:** 2016-11-08

**Authors:** Benjamin Kelmendi, Thomas G. Adams, Stephanie Yarnell, Steven Southwick, Chadi G. Abdallah, John H. Krystal

**Affiliations:** 1 Department of Psychiatry, Yale University School of Medicine, New Haven, CT; 2 Clinical Neuroscience Division, Department of Veterans Affairs, National Center for Posttraumatic Stress Disorder, Veterans Affairs Connecticut Healthcare System, West Haven, CT; 3 Abraham Ribicoff Research Facilities, Connecticut Mental Health Center, New Haven, CT

**Keywords:** PTSD, noradrenergic, serotonin, GABA, cannabinoid, ketamine, glutamate, pharmacology

## Abstract

Posttraumatic stress disorder (PTSD) is a chronic debilitating psychiatric disorder characterized by symptoms of re-experience, avoidance, and hyperarousal that can arise immediately or many years after exposure to a traumatic event and injury. Although extensive research has been done over the past 30 years, the etiology of PTSD remains largely unknown. Several neurobiological systems have been implicated in the pathophysiology and vulnerability for developing PTSD; however, first-line pharmacotherapies are limited. Less than 30% achieve full remission, and even then, approved pharmacological treatments often take weeks for therapeutic effect. This article aims to review the pathophysiology of PTSD within multiple neurobiological systems and how these mechanisms are used as pharmacologic targets of treatment, as well as their potential for future targets of intervention.

**Highlights of the article:**

- We reviewed the neurobiological abnormalities in PTSD as they relate to well-established, preliminary, and future targets for pharmacological interventions.
- Abnormalities across different neurotransmitter systems have been implicated in the pathophysiology of PTSD but none of these systems function uniformly among all patients with PTSD
- First-line pharmacotherapy for PTSD provides a suboptimal response rates.
- Future pharmacological targets for PTSD include the cannabinoid and oxytocin systems, as well glutamatergic modulating agents.
- Drug development for PTSD should specifically address various dimensions of PTSD symptomatology.

Posttraumatic stress disorder (PTSD) is a common, chronic, and disabling condition characterized by symptoms of re-experiencing, avoidance, and hyperarousal following traumatic experiences (e.g., military combat, a natural disaster, and/or physical or sexual assault) that are distinct from ordinary life stressors. Symptoms may develop immediately or years after exposure. PTSD is a heterogeneous disorder; the occurrence of symptoms is not predictable, the disorder may not present with the same constellation of symptoms in each afflicted person, and many times, the initial presentation of the disorder is confounded by other psychiatric comorbidities. Lifetime prevalence rates of PTSD in the general population range between 6.4 and 7.8% (Pietrzak, Goldstein, Southwick, & Grant, 2011), and approximately 20% among combat-exposed military veterans (Seal et al., 2009).

The only two FDA-approved pharmacological treatments for PTSD, paroxetine and sertraline, rarely produce a response rate exceeding 60%, and less than 30% of the patients achieve clinical remission (Berger et al., 2009). In several recent placebo-controlled studies of alternate medications for PTSD, medications did not perform better than placebo (Pitman et al., 2012). Given sub-optimal treatment, additional research is needed to investigate the basic mechanisms and underlying pathways implicated in this disorder. This article reviews current understanding of a number of interrelated neurotransmitter systems that have been implicated in the mediation of stress response, dissociative symptoms, formation of traumatic memories, and the pathophysiology of PTSD with emphasis placed on the catecholamines, glutamatergic, gamma-aminobutyric acid (GABA) ergic systems, and cannabinoids, among others. Through analysis of underlying neurobiological mechanisms implicated in the pathophysiology of PTSD, we will review potential treatment targets and discuss future research directions.

## Serotonin

### Neurobiology

The cell bodies of the serotonin (5-HT) neurotransmitter system are located in brainstem's median and dorsal raphe nuclei, which project widely in the brain, including to key fear circuitry loci within the amygdala, hippocampus, and ventromedial prefrontal cortex (vmPFC), and primarily target GABAergic inhibitory neurons (Neumeister et al., 2013). Numerous preclinical studies have reported heightened 5-HT release, enhanced neuronal activity in the dorsal raphe nuclei, and increased 5-HT synthesis and turnover in response to acute stress (Krystal & Neumeister, 2009). Furthermore, administration of meta-chlorophenylpiperzine (mCPP), a 5-HT_2C_ receptor agonist, resulted in acute anxiety, panic attacks, and PTSD symptoms in a subgroup of male combat veterans with PTSD, but not other psychiatric disorders, suggesting a role of the 5-HT system in the pathophysiology of PTSD (Krystal et al., 1996; Krystal & Neumeister, 2009). Pharmacological agents that enhance serotonergic activity, such as 5-HT reuptake inhibitors (SSRIs) that block the 5-HT transporter, are partially efficacious in treating PTSD symptoms (Berger et al., 2009). However, considering that mCPP induces panic attacks and dissociative symptoms in patients with PTSD, it is surprising that the initial increased 5HT concentration observed with SRIs does not exacerbate PTSD symptoms. Recent study has shed light on this complex issue by showing that mCPP can also produce panic attacks and dissociative symptoms in health controls when pre-treated with iomazenil, inverse GABA agonist (D'Souza et al., 2006). These findings suggest that the deficits in GABAergic inhibition perhaps resulting from stress-related serotonin and norepinephrine (NE) input may contribute to the dissociative effects of mCPP in patients with PTSD.

Clinical and preclinical studies have implicated stimulation and interaction of 5-HT_1A_, 5-HT_1B_, and 5-HT_2A_ or 5-HT_2C_ receptors in antidepressant and/or anxiolytic action in PTSD (Neumeister et al., 2013), but this emphasis may, in part, be an artifact related to the availability of ligands selected for these receptor subtypes. Disruption of 5-HT_1A_ expression in forebrain during development may result in a lifelong anxious phenotype (Neumeister et al., 2013). Preclinical evidence has shown 5-HT_1A_ receptor knockout mice demonstrate increased anxiety and fear response (Ramboz et al., 1998), suggesting that 5-HT_1A_ agonist may have therapeutic implications in PTSD. Similarly, a positron emission tomography (PET) study found higher 5-HT_1A_ binding (26–43%) in PTSD compared with healthy controls in every region of interest examined, except hippocampus, with the highest concentration found in raphe nuclei (Sullivan et al., 2013).

The 5-HT_1B_ receptor may be of particular relevance to PTSD. Serotonin, via 5-HT_1B_ receptors, modulates cortical inhibitory input onto subcortical structures. In animals, stress exposure reduces 5-HT_1B_ receptor function in multiple brain regions, resulting in behaviors resembling chronic anxiety (Sari, 2004). The caudate, amygdala, and anterior cingulate cortex (ACC) have all been implicated in PTSD and were shown by PET to have markedly reduced 5-HT_1B_ receptor density among PTSD patients (Murrough et al., 2011). Thus, 5-HT_1B_ receptors appear to play a critical role in the modulation of fear and anxiety (Neumeister et al., 2013).

### Pharmacological interventions

Double-blind controlled trials demonstrate paroxetine and sertraline improve PTSD symptomatology (Alexander, 2012). Previous trials have demonstrated the superiority of paroxetine over placebo in managing PTSD symptoms (Alexander, Lund, Bernardy, Christopher, & Friedman, 2015). Paroxetine demonstrated significant superiority to placebo regarding improvement from baseline in the Clinician-Administered PTSD Scale (CAPS-2) total score, but not for the proportion of responders on the Clinical Global Impression-Improvement (CGI-I) Scale (Marshall, Beebe, Oldham, & Zaninelli, 2001). Similarly, when 187 individuals with chronic PTSD were randomly assigned to receive sertraline or matched placebo, sertraline provided a significantly greater improvement in primary outcome measures with response rates of 53 and 32%, respectively (Brady et al., 2000). Another 12-week double-blind study of 208 patients with moderate-to-severe PTSD randomly assigned individuals to sertraline and placebo (Davidson, Rothbaum, van der Kolk, Sikes, & Farfel, 2001); sertraline was found to provide a significantly greater improvement on all primary outcome measures with response rate 60%, and the response rate was 38% for placebo. However, in contrast to the studies that demonstrated efficacy for SSRIs in PTSD, clinical studies for combat-related PTSD, involving veterans seeking treatment in Department of Veterans Affairs (VA) hospital, have shown mixed results. In a double-blind placebo-controlled 10-week study of 42 military veterans with combat-induced PTSD, sertraline resulted only in non-statistically significant improvement (Zohar et al., 2002). In another double-blind placebo-controlled study, 12 weeks of flexible doses of sertraline (25–200 mg/day) did not demonstrate to be efficacious in the treatment of PTSD in 196 patients recruited from 10 different VA medical centers (Friedman, Marmar, Baker, Sikes, & Farfel, 2007). Given these findings, unsurprisingly, recent guidelines on the treatment of PTSD question the recommendation of SSRIs for veterans with combat-related PTSD (Benedek, Friedman, Zatzick, & Ursano, 2009).

The first double-blind placebo-controlled trial of antidepressants [phenelzine, a monoamine oxidase inhibitor, and imipramine, a tricyclic antidepressant] showed both medications to be superior to placebo in patients with PTSD (Frank, Kosten, Giller, & Dan, 1988). However, due to the side effects associated with these classes of antidepressants, they are less commonly prescribed in clinical practice. In a 12-week double-blind study, extended-release (ER) venlafaxine, a serotonin–norepinephrine reuptake inhibitor (SNRI), as well as sertraline was performed in patients with PTSD (Davidson et al., 2006). A total of 538 subjects were randomly assigned to receive venlafaxine ER, sertraline, or placebo. Of the 350 who completed the study, remission rates were 30.2% for venlafaxine ER (*P*<0.05 vs. placebo), 24.3% for sertraline, and 19.6% for placebo. In another study, 6 months of flexible doses of venlafaxine ER resulted in greater rates of remission (50.9%) compared with placebo (37.5%) (Davidson et al., 2006). In both studies, venlafaxine ER failed to improve hyperarousal symptoms.

3,4-methylenedioxy-methamphetamine (MDMA), also identified as the street drug “ecstasy,” has been shown to induce the release of serotonin (Liechti & Vollenweider, 2001) and NE, producing psychostimulant effects in humans (Hysek et al., 2011). Pilot studies suggest that MDMA can be safely administered to patients and that MDMA-assisted therapy appears to facilitate the emergence of fearful memories that can then be reprocessed in therapy, without intensely negative emotions (Mithoefer, Wagner, Mithoefer, Jerome, & Doblin, 2011). However, negative mood states and psychobiological distress reactivity have been associated with MDMA (Mithoefer et al., 2013). Also, there is substantial abuse liability. Therefore, further research and randomized controlled trial (RCT) are required to explore both the positive and negative effects of MDMA.

In summary, while SSRIs are the most commonly prescribed pharmacological agents for the treatment of PTSD, as they are relatively well tolerated and safe, they suffer a number of shortcomings. The onset of the therapeutic effect takes several weeks; their utility is further limited by a common partial response with troubling residual symptoms, or non-response, as well as persistent undesirable side effects such as changes in appetite and weight, gastrointestinal disturbances, and loss of sexual drive.

## Noradrenergic system

### Neurobiology

The prominence of hyperadrenergic symptoms in PTSD (e.g., hyperarousal, re-experiencing, anxiety, tachycardia, and diaphoresis) made NE a focus point of investigation (O'Donnell, Hegadoren, & Coupland, 2004). Evidence for an overactive noradrenergic system in PTSD comes from the investigation of neuroendocrine and peripheral catecholamine (epinephrine, NE, and dopamine), transporter, and receptor systems (Southwick, Morgan, et al., 1997). Early studies found abnormally high levels of catecholamines and their metabolites in the plasma and urine of individuals undergoing severe stress, as well as patients with PTSD, suggesting increased levels of catecholamines may be responsible for certain PTSD symptoms (Southwick et al., 1999).

Studies examining the number of alpha-2-adrenergic receptor sites have shown that both combat veterans and children diagnosed with PTSD had fewer alpha-2-adrenergic receptor-binding sites per platelet than did healthy controls (Perry, Giller, & Southwick, 1987; Pitman et al., 2012), suggesting putative adaptive changes in response to the higher levels of circulating catecholamines. Further evidence for the involvement of NE in PTSD comes from pharmacological challenge studies inducing sympathetic nervous system activation with yohimbine, an alpha-2-adrenergic auto- and post-synaptic receptor antagonist, which increases synaptic NE by blocking autoinhibitory feedback (Southwick, Krystal, et al., 1997). Administration of yohimbine decreased metabolic activity in the prefrontal cortex (PFC) and resulted in panic attacks and flashbacks for 70 and 40%, respectively, of the veterans with PTSD (Southwick, Krystal, et al., 1997). Interestingly, administration of yohimbine in patients with schizophrenia, major depression disorder, generalized anxiety disorder, or obsessive–compulsive disorder does not produce similar effects (Krystal & Neumeister, 2009).

Dysregulation of norepinephrine transporter (NET) expression may result from hyperadrenergic states. Preclinical studies show that endogenous dopamine and NE stimulate NET expression in the central and peripheral nervous system (Krystal & Neumeister, 2009). The NET has a high concentration in the LC and moderate levels in the PFC, hippocampus, amygdala, and thalamus (Berman et al., 2000). It acts as NE plasma membrane transporter and maintains presynaptic NE storage. Chronic stress leads to a reduction of NET availability in LC, while in the PFC there is an increase in NET expression, suggesting this may represent an attempt to maintain normal availability, and consequently normal function of NE (Pietrzak et al., 2013). These preclinical findings were recently replicated in a human PET study demonstrating that PTSD is associated with significantly decreased NET availability in LC, which might, in turn, result in the exaggerated synaptic availability of NE in projection areas, such as the PFC (Pietrzak et al., 2013). Despite these informative preclinical models and clinical evidence, the role of the antidepressants with a high affinity for NETs in the treatment of PTSD remains unclear.

Stress-induced reductions in neuropeptide Y (NPY), a protein known to inhibit NE release, are also implicated in overall increased release of NE in PTSD (Neumeister et al., 2013; Perry et al., 1987). With its highest concentration in LC, hypothalamus, septum, and periaqueductal gray, NPY is implicated in arousal and the assignment of emotional valences to stimuli and memory (Silva, Xapelli, Grouzmann, & Cavadas, 2005). Human research suggests NPY confers anxiolytic effects and is also involved in stress resilience (Morgan et al., 2002). Soldiers in the Special Forces who underwent extremely stressful training programs had higher NPY levels compared with non-Special Force soldiers and were subsequently found to have better performance during training and lower stress-induced dissociation (Morgan et al., 2000). Compared with healthy controls, patients with PTSD were shown to have lower baseline plasma NPY levels and a blunted yohimbine-induced NPY increase, suggesting susceptibility of the system to a pharmacological stressor (Morgan et al., 2000; Morgan et al., 2002). Pretreatment with intranasal NPY has been shown to attenuate the development of PTSD-like symptoms in rodent models of PTSD (Sabban, Alaluf, & Serova, 2015). However, efforts to develop pharmacological agents harnessing NPY-receptor-mediated effects have thus far been unsuccessful (Pitman et al., 2012).

### Pharmacological interventions

Sustained hyperadrenergic activity at night has been associated with poor sleep and nightmares. Prazosin, a post-synaptic alpha-1-noradrenergic receptor inhibitor, has been shown to increase sleep duration and decrease trauma-related nightmares in PTSD (Raskind et al., 2013). A recent study of 22 veterans with PTSD found prazosin was effective in treating nightmares and non-nightmare distressed awakenings (Raskind et al., 2013; Taylor et al., 2008). This evidence suggests that prazosin may be a promising adjunctive medication to target sleep-related disturbances in patients with PTSD (Petrakis et al., 2016).

Desipramine may also help with PTSD symptomatology. A recent double-blind study compared the efficacy of desipramine, an inhibitor of NE reuptake, with paroxetine in PTSD patients with comorbid alcohol use disorder (Petrakis et al., 2012). This study also evaluated the adjunctive efficacy of naltrexone relative to placebo. Patients were assigned to one of four groups: paroxetine +/− naltrexone and desipramine +/− naltrexone. Desipramine was found to be superior to paroxetine on study retention and alcohol use outcomes; paroxetine did not show statistical superiority to desipramine for the treatment of PTSD (Petrakis et al., 2012).

## Glutamatergic system

### Neurobiology

Glutamate as the primary excitatory neurotransmitter in the CNS is implicated in the rapid processing of information between and throughout cortical and subcortical structures (Chambers et al., 1999). The catecholamine systems, as well as local inhibitory neurons using GABA, converge on pyramidal neurons, which is the primary source of glutamate in the cortex (Chambers et al., 1999). Thus, glutamate transmission is integral to many CNS processes of learning, memory, and plasticity. The glutamatergic system utilizes receptors that are generally classified into two categories: ionotropic and metabotropic. Ionotropic glutamate receptors have ion channels and allow for rapid synaptic transmission; they consist of *N*-methyl-D-aspartate receptor (NMDA), α-amino-3-hydroxy-5-methyl-4-isoxazolepropionic acid (AMPA), and kainate receptors. The glutamate system also consists of three groups of metabotropic receptors, which are coupled to secondary messengers.

Increasing evidence suggests the involvement of glutamatergic system in pathophysiology of PTSD (Pitman et al., 2012). The main projections from PFC to the amygdala or to the other neurotransmitter inputs into the amygdala are glutamatergic in nature (Del Arco & Mora, 2009). Acute stress exposure in rats induces increases in glutamate transmission across different brain regions such as PFC, amygdala, and hippocampus (Pitman et al., 2012). Not surprising abnormal glutamate levels in PFC and NMDA receptor density in hippocampus have been reported in animal models of PTSD (Pitman et al., 2012). Perturbations in the glutamatergic system have also been replicated in human population. In a recent magnetic resonance spectroscopy (MRS) study, patients with PTSD compared with trauma-exposed controls were shown to have elevated glutamate levels in the lateral temporal cortex and lower levels in ACC in PTSD with alcohol use disorder (Pennington, Abe, Batki, & Meyerhoff, 2014). Elevated serum glutamate levels were recently reported in patients with PTSD 3 months after a traumatic accident compared with healthy controls (Nishi et al., 2015). The insufficient top-down control from the PFC activation to the amygdala implicated in the pathophysiology of PTSD suggests, direct or indirect, involvement of glutamatergic transmission. This is further illustrated by examining the involvement of glutamate receptors in PTSD symptomatology, most notably the NMDA receptor. Although metabotropic glutamate receptors have also been implicated in stress-related pathology, much of the work is isolated to preclinical models of anxiety and more conclusive work remains to be done in human clinical studies.

Glutamatergic transmission is implicated in the pathophysiology of PTSD via the crucial role the NMDA receptors play in synaptic plasticity underlying learning and memory (Chambers et al., 1999). More specifically, NMDA receptors have shown to be involved in fear conditioning; therefore, NMDA agonist could facilitate fear extinction in patients with PTSD (Bailey, Cordell, Sobin, & Neumeister, 2013). On the contrary, administration of NMDA receptor antagonist such as ketamine and PCP to healthy controls has been shown to produce dissociative symptoms similar to that reported in PTSD (Bailey et al., 2013). Local injection of subanesthetic doses of ketamine in rat brains has been shown to increase glutamate efflux in PFC (Moghaddam, Adams, Verma, & Daly, 1997), which make NMDA antagonists important targets for studying the link between glutamate system and dissociative symptomatology.

### Pharmacological interventions

Ketamine is a glutamate NMDA-R antagonist that is commonly used as an anesthetic. Psychiatric interest in ketamine has grown following the discovery of ketamine's rapid antidepressant effects (Berman et al., 2000). Preclinical studies have demonstrated that subanesthetic doses of ketamine increase glutamate release, brain-derived neurotrophic factor (BDNF) signaling, and concomitant stimulation of neurogenesis and synaptogenesis (Abdallah, Sanacora, Duman, & Krystal, 2015).

Early case series suggested that anesthetic doses of ketamine might reduce posttraumatic stress symptoms among burn victims receiving surgery (McGhee, Maani, Garza, Gaylord, & Black, 2008). Conversely, peritraumatic administration of anesthetic ketamine before surgery was associated with a worsening of acute posttraumatic stress symptoms among burn victims (Schonenberg, Reichwald, Domes, Badke, & Hautzinger, 2005, 2008). These studies suggest that anesthetic doses of ketamine might potentiate memory if administered shortly following a traumatic event and may reduce symptomatology if administered well after the traumatic event. A recent case report suggested that a single subanesthetic dose of ketamine may temporarily reduce posttraumatic stress symptoms (D'Andrea & Andrew Sewell, 2013). Only one randomized controlled trial has tested the effects of ketamine on PTSD (Feder et al., 2014); ketamine and midazolam were administered to 41 patients with a primary diagnosis of PTSD in a randomized, double-blind, crossover trial. Both drugs were associated with rapid reductions in posttraumatic stress symptoms, but ketamine outperformed midazolam.

D-Cycloserine (DCS) is a partial NMDA receptor agonist that has been tested as a monotherapy and treatment augmentation agent for PTSD. There is minimal evidence that DCS is efficacious as monotherapy (Heresco-Levy et al., 2002) or as an add-on for standard pharmacological treatments for PTSD (Attari, Rajabi, & Maracy, 2014). There is weak evidence that DCS can enhance the efficacy of exposure therapy for PTSD. Only one RCT has found that PTSD patients who receive DCS and exposure therapy fare better than their counterparts who receive exposure therapy with a placebo pill (Difede et al., 2014). Two other RCTs failed to show therapeutic exposure enhancing effects of DCS (de Kleine, Hendriks, Kusters, Broekman, & van Minnen, 2012; Rothbaum et al., 2014). One study reported that DCS was detrimental (Litz et al., 2012). The only study to report positive effects of DCS used a larger dose (100 mg) than those studies that reported negative findings (50 mg) (Difede et al., 2014), raising the possibility that larger doses are required in PTSD to significantly enhance exposure therapy.

## GABA

### Neurobiology

Disturbances in the GABAergic system have been detected in PTSD. Studies have shown significantly lower GABA levels in both the mesial temporal lobe and the parieto-occipital cortex among individuals exposed to trauma who developed PTSD as compared to those who did not (Meyerhoff, Mon, Metzler, & Neylan, 2014). GABA has previously been shown to play an important role in memory registration and emotional and fear memory encoding (Corcoran & Maren, 2001). Reduced GABA tone in the parieto-occipital cortex strongly correlated with severity of insomnia symptoms (Meyerhoff et al., 2014). Decreased GABA levels in PTSD are consistent with previous finding of low cortical and subcortical benzodiazepine receptor binding in patients with PTSD and panic disorder (Bremner, Southwick, Darnell, & Charney, 1996; Charney, 2004) and may suggest GABA as a potential therapeutic target.

### Pharmacological interventions

Treatment of PTSD-related symptoms has long included GABAergic targets. Benzodiazepines are a very popular form of treatment for PTSD, but their use is in decline (Lund, Bernardy, Alexander, & Friedman, 2012) due to addictive potential. While helpful for insomnia and anxiety (Lund et al., 2012), benzodiazepines are not effective for avoidance and dissociation (Viola et al., 1997), impair fear extinction (Rothbaum et al., 2014), and can reduce the effectiveness of exposure therapy (van Minnen, Arntz, & Keijsers, 2002). The 2010 VA/DoD Clinical Practice Guideline discouraged the use of benzodiazepines for the treatment of both acute stress disorder and PTSD, citing evidence that risks outweigh benefits and that benzodiazepines might worsen recovery from trauma (VA/DoD, 2010). Eszopiclone – a high affinity GABA-A receptor agonist – has been shown to significantly improve sleep disturbance associated with PTSD (Pollack, Jensen, Simon, Kaufman, & Renshaw, 2008). GABAergic anticonvulsants are also often prescribed for PTSD-related symptoms, but results have not been uniform. One large placebo-controlled study of tiagabine found no significant effect of the drug on PTSD, depression, or functional impairment (Davidson, Brady, Mellman, Stein, & Pollack, 2007). Divalproex was similarly shown to have no significant effect on PTSD symptoms (Hamner et al., 2009). In a small international trial, topiramate was reported to significantly reduce PTSD symptoms (Yeh et al., 2011), but these results have yet to be replicated. In summary, GABAergic drugs are widely prescribed but incompletely understood, and clinical trials have thus far yielded underwhelming results. Each comes with potential side effects, tolerance issues, and addictive potential, as well as possible effects on neurocognitive functioning.

## Cannabinoids

### Neurobiology

Cannabinoid receptor 1 (CB1) is the most abundant receptor in the central nervous system (Glass, Dragunow, & Faull, 1997; Herkenham et al., 1990) and is found in highest concentrations in the amygdala–hippocampal–corticostriatal (AHC) circuit, an area responsible for coordinating fear-related behaviors, as well as storing and processing fear-related memories (LeDoux, 2000; Rogan, Staubli, & LeDoux, 1997). CB1 receptors are involved in consolidation and extinction of aversive memories; glucocorticoid-hormone facilitated potentiation of NE in fear conditioning, and extinction circuitry is mediated by CB1 receptors (Atsak, Roozendaal, & Campolongo, 2012; Campolongo et al., 2009; Hill & McEwen, 2009; Marsicano et al., 2002; Roozendaal, Barsegyan, & Lee, 2008). It has been demonstrated that CB1 receptor availability is higher in individuals with PTSD (Neumeister, 2013); the highest CB1 availability was in the AHC, implicating a compensatory up-regulation of CB1 receptors (Neumeister, 2013). An associated lowering of endogenous CB1 agonist, anandamide (ANA), was similarly found in PTSD (Neumeister, 2013). Augmentation of ANA has been shown to modulate short-term fear extinction in animals (Pamplona, Bitencourt, & Takahashi, 2008), resulting in a long-term reduction in fear (Gunduz-Cinar et al., 2013). Together, research suggests the endocannabinoid system may be a potential target system for PTSD (Neumeister, 2013; Neumeister, Seidel, Ragen, & Pietrzak, 2015).

### Pharmacological interventions

While no large-scale trials have been completed, small studies support the use of cannabis for PTSD. One uncontrolled, cross-sectional, retrospective self-report study found that individuals with significant posttraumatic stress symptoms reported that their symptoms were 75% less severe when they were using cannabis compared with when they were not (Greer, Grob, & Halberstadt, 2014). Cannabis has been reported to be particularly helpful to persons with severe traumatic intrusions (Bonn-Miller, Boden, Bucossi, & Babson, 2014) and has been shown to help manage hyperarousal symptoms (Bremner et al., 1996). However, while these small studies are of benefit, they do not carry the same weight as randomized control trials leaving the degree of potential benefit in question.

Use of cannabis as treatment is complicated. First, there has long been the concern for impairing cognition, as well as paranoia (Yarnell, 2015). Second, there is a concern for potential long-term worsening of PTSD outcomes with cannabis (Wilkinson, Yarnell, Radhakrishnan, Ball, & D'Souza, 2016). Marijuana use is positively correlated with PTSD symptoms, but, according to self-reports, cannabis was used with intent to cope with these PTSD symptoms (Bonn-Miller, Vujanovic, Feldner, Bernstein, & Zvolensky, 2007). Therefore, the directionality of the effects of cannabis use on PTSD symptoms cannot be fully differentiated (Yarnell, 2015). Regardless, there appears also to be a correlation between PTSD and problematic cannabis use (Wilkinson et al., 2016; Yarnell, 2015). Third, extended use may result in down-regulation of CB1 receptors (Leweke & Koethe, 2008), predisposing to a rebound anxious/depressive phenotype (Haller, Bakos, Szirmay, Ledent, & Freund, 2002; Haller, Varga, Ledent, & Freund, 2004).

To address some of these concerns, some have suggested targeting fatty acid amide hydrolase (FAAH), the enzyme that degrades endocannabinoids (Varvel, Wise, Niyuhire, Cravatt, & Lichtman, 2007). When the endocannabinoid system was activated through slowing the breakdown of endogenous cannabinoids, animal models demonstrated improved extinction of aversive memories (Lutz, 2007). This finding is consistent with other studies that have shown cannabinoids can facilitate fear extinction learning without affecting positive memory formation (Chhatwal, Davis, Maguschak, & Ressler, 2005; Marsicano et al., 2002). Others have suggested using CB1 antagonists (Yarnell, 2015). However, cannabinoid-deficient mice required significantly longer time to forget an association with painful foot shocks and bell ringing than wild-type control (Chhatwal et al., 2005; Lutz, 2007; Marsicano et al., 2002) suggesting antagonism may not be beneficial. Given the mixture of concern and potential results, future efforts may focus on replacement of specific endocannabinoids.

## Oxytocin

Oxytocin (OT) has recently received a great amount of interest in treatment of psychological disorders. Disruption in the OT system has been implicated in the development of PTSD (Feldman, Vengrober, & Ebstein, 2014). OT is well known for its pro-social effects and anxiolytic properties and it has been suggested as promising psychological intervention to enhance treatment response in PTSD (Olff et al., 2014).

Studies in humans have provided evidence that OT administration does not affect fear conditioning but has the potential to facilitate fear extinction and attenuate fear response (Heinrichs, Baumgartner, Kirschbaum, & Ehlert, 2003). Imaging studies in humans have found that intranasal OT has the ability to dampen amygdala activity in response to threatening visual stimuli (Koch et al., 2014, 2016). These imaging studies suggest that patients with PTSD with high anxiety and high baseline amygdala reactivity may especially benefit from OT administration; in other words, by dampening excessive fear processing during exposure-based therapies, OT administration could result in enhanced treatment response. In summary, it is possible that OT could either be used alone or in conjunction with other therapies such as exposure therapy or could also possibly be used as prophylactic. However, effects of OT administration remain to be thoroughly investigated across different clinical settings before considering routine clinical application.

## Conclusion

Currently, FDA-approved treatments have demonstrated mild-to-moderate success. The research reviewed in this article reveals that while much knowledge has been generated toward the underlying pathophysiology of PTSD and many pharmacological treatments have emerged, very few research results have resulted in novel, effective treatments. The currently approved treatments have demonstrated mild-to-moderate success, and most novel treatments have insufficient evidence to draw meaningful conclusions about their efficacy. This article reviewed manipulations of the glutamate system via ketamine, cannabinoids such as FAAH inhibitors and medical marijuana, and DCS all of which could potentially be used as cognitive enhancers. One possible treatment approach for this complex disorder may be the use of multiple drugs simultaneously due to the heterogeneity of the PTSD phenotype. When determining the appropriate drug or drugs to be prescribed, clinicians should consider which symptom clusters are most prevalent and severe in an individual. Another promising avenue for future research direction, which gained increasingly more attention, is to identify target systems involved in fear extinction and explore interventions that enhance targeted approach of exposure-based psychotherapies for PTSD such as DCS, oxytocin, and MDMA (Heinrichs et al., 2003; Young, Andero, Ressler, & Howell, 2015). There is also some evidence that morphine shortly after exposure to traumatic events reduces the likelihood to develop PTSD (Bryant, Creamer, O'Donnell, Silove, & McFarlane, 2009; Saxe et al., 2001). However, while these small studies are of benefit, they do not carry the same weight as randomized control trials leaving the degree of potential benefit in question.
